# Influence of Allyl Isothiocyanate on the Soil Microbial Community Structure and Composition during Pepper Cultivation

**DOI:** 10.4014/jmb.2012.12016

**Published:** 2021-05-29

**Authors:** Jingxia Gao, Hongxia Pei, Hua Xie

**Affiliations:** Institute of Germplasm Resources, Ningxia Academy of Agriculture and Forestry Sciences, Yinchuan 750002, P.R. China

**Keywords:** Pepper, allyl isothiocyanate, enzyme activity, microbial diversity, microbial community

## Abstract

Allyl isothiocyanate (AITC), as a fumigant, plays an important role in soil control of nematodes, soilborne pathogens, and weeds, but its effects on soil microorganisms are unclear. In this study, the effects of AITC on microbial diversity and community composition of *Capsicum annuum* L. soil were investigated through Illumina high-throughput sequencing. The results showed that microbial diversity and community structure were significantly influenced by AITC. AITC reduced the diversity of soil bacteria, stimulated the diversity of the soil fungal community, and significantly changed the structure of fungal community. AITC decreased the relative abundance of dominant bacteria *Planctomycetes*, *Acinetobacter*, *Pseudodeganella*, and *RB41*, but increased that of *Lysobacter*, *Sphingomonas*, *Pseudomonas*, *Luteimonas*, *Pseudoxanthomonas*, and *Bacillus* at the genera level, while for fungi, *Trichoderma*, *Neurospora*, and *Lasiodiplodia* decreased significantly and *Aspergillus*, *Cladosporium*, *Fusarium*, *Penicillium*, and *Saccharomyces* were higher than the control. The correlation analysis suggested cellulase had a significant correlation with fungal operational taxonomic units and there was a significant correlation between cellulase and fungal diversity, while catalase, cellulose, sucrase, and urease were the major contributors in the shift of the community structure. Our results will provide useful information for the use of AITC in the assessment of environmental and ecological security.

## Introduction

With the rapid development of the vegetable industry in China, the vegetable yield of protective agriculture methods, such as greenhouse cultivation, has increased rapidly [1]. *Capsicum annuum* L., or pepper, is rich in polyphenols, capsaicin, ascorbic acid, and plant chemicals [2], and has antibacterial, antioxidant, weight control, and other functional characteristics [3, 4]. Pepper is one of the most widely cultivated vegetables in China. Therefore, improving the yield and quality of pepper is of great significance to increase farmers’ income and increase vegetable exports.

Pepper has a large cultivated area in the mountainous region in southern Ningxia, China. However, due to the restriction of local cultivated land and the demand for high yields, the phenomenon of the continuous cropping of vegetables in protective agriculture is relatively common, which leads to imbalances in the soil microbial community and an increase in diseases and insect pests [5-7]. Reducing the population of beneficial microorganisms and increasing soil-borne pathogens will eventually give rise to the outbreak of soil-borne diseases in pepper and the reduction of crop production [8, 9]. Thus, one of the main factors affecting crop yield and quality is the spread of soil diseases [10, 11]. Soil disinfection, such as fumigation, is one of the simplest, most economic and effective technical measures to prevent and control soil-borne diseases [12-14]. At present, methyl bromide (MB), chloropicrin (CP), azomet (DZ), 1,3-dichloropropene (1,3-D), allyl isothiocyanate (AITC), metasodium (MS), and dimethyl disulfide (DMDs) have been widely used in soil fumigation [15]. Among them, AITC, commonly known as horseradish, can control soil insects, pathogens, nematodes, and weed seeds effectively [16, 17], and has the characteristics of broad spectrum, high efficiency, and environmental friendliness. The use of AITC for the prevention and control of plant pathogenic fungi, bacteria, nematodes, and other diseases has gradually increased [18].

Soil microorganisms include bacteria, fungi, and actinomycetes [19], all of which not only participate in nutrient cycling, organic matter decomposition, and the transformation of carbon, nitrogen, and other elements in the soil, but also reflect the health status of the soil [20], which is believed to be responsible for the biological processes necessary to maintain healthy soil and inhibit plant diseases [21]. The diversity of the soil microbial population or changes in the dominant flora can reflect the development of soil and further affect the growth and development of crops [22, 23]. Therefore, soil fertility can be judged according to changes in the soil microorganisms, which not only is of great significance to the ecological environment [24], but also plays an important role in maintaining ecological balance [25]. Therefore, it is of great significance to study soil microorganisms for agricultural production. However, the biochemical and material cycling processes of soil cannot be separated from the participation of soil enzymes, and soil fertility and microbial activity are also closely related to soil enzymes [26]. Soil enzymes are an important driving force of material and energy cycling and must be considered to evaluate the soil microbial community ecosystem and overall soil health [27]. Soil microorganisms are an important source of soil enzymes, and the activity of soil microorganism can reflect the level of enzyme activity in soil [28]. Research has shown that when the number of soil microorganisms increases, microbial secretions also increase, leading to a rise in soil enzyme activity [29]. With the development of molecular biology, metagenome sequencing and fluorescence quantitative PCR or conventional biological methods have been applied to study the effects of fumigants on the soil microbial community, population diversity, dominant flora, and nitrogen cycle functional microorganisms [30, 31]. Gaining an understanding of the effects of different fumigants on soil microorganisms is not only related to the recovery of soil microorganisms after fumigation, but also related to the yield and quality of crops planted in fumigation plots. Fang *et al*. used real-time quantitative PCR (qPCR) and 16S rRNA gene amplicon sequencing techniques to determine that fumigant 1,3-D significantly affected the nitrogen cycling microorganisms in the soil [32]. Huang *et al*. [33] found that CP fumigation stimulated an increase in the relative abundance of microorganisms involved in carbohydrate/lipid transport and metabolism functions after fumigation. Fang *et al*. [34] reported that DZ fumigation significantly reduced the abundance of 16S rRNA and nitrogen cycling functional genes and caused significant changes in soil bacterial diversity and community composition. MS also caused a long-term significant decline in the bacterial community diversity and changed the bacterial community structure [35].

AITC, as an insecticide, not only protects crop production by controlling soil-borne pathogens, but also affects soil microbial diversity and community composition [36]. Zhu *et al*. [1] found that AITC had less effects on bacterial than fungal communities and temporarily reduced the diversity of soil bacteria while stimulated the diversity of soil fungi in the long term and significantly altered the structure of the fungal community. In addition, other studies found that the relative abundance of soil bacterial communities changed after fumigation with AITC: at the phylum level, the relative abundance of Proteus bacteria increased significantly in the short term, while the relative abundance of sulfur bacteria decreased significantly in the short term; among them, the relative abundance of *Firmicutes* increased significantly, which may be related to its strong stress resistance [37, 38]. Fang *et al*. [34] found that although some phyla increased or decreased, DMDs and AITC fumigation had no significant effects on the composition and structure of the main soil microbial communities. Proteobacteria, Chloroflexi, and Acidobacteria decreased significantly, while *Firmicutes*, Gemmatimonadetes, Actinobacteria, Verrucomicrobia, Saccharibacteria, and Parcubacteria increased. Wang *et al*. found that fumigation had a significant effect on microbial community structure [39]. However, some studies reported that fumigation had little effect on the abundance and diversity of soil microbial species, but affected the community structure to a certain extent [12].

Although AITC can effectively control soil-borne diseases, to date there have been few studies focusing on the response of soil microorganisms to AITC fumigants, especially on the diversity and structure of soil bacterial and fungal communities. In order to clarify the effect of AITC fumigation on microorganisms in pepper soil and ensure the quality and safety of pepper soil, we combined the Hiseq2500 PE250 high-throughput sequencing platform with related bioinformatics to analyze the richness and diversity of the 16S rRNA gene V3–V4 region of soil bacteria and the ITS2 region of fungi after DNA extraction, PCR amplification, and database construction. This research provides a theoretical basis for the environmental safety assessment of AITC and its impacts on soil microorganisms.

## Materials and Methods

### Site Description

The experiment was conducted at the Arched Shed Pepper Core Demonstration Base in Xinji Township, Pengyang County, Ningxia (E 106°38’, N 35°51’) (continuous cropping of pepper for 8 years). The region is a temperate semi-arid area, with a typical continental monsoon climate of large daily temperature differences and sufficient light. The annual average temperature, the frost-free period, and the annual average precipitation are 7.2°C, 140–170 days, and 350–550 mm. The landform type is the hilly and gully area in the middle of the Loess Plateau with an altitude of 1,286–2,416 m. The soil type is black loessial soil, in which the organic matter content and other nutrients are high. The soil management followed standard practices for the area and the basic parameters of soil were as follows: pH 8.37, available N 78.2 mg·kg^-1^, available P 18.3 mg·kg^-1^, available K 406 mg·kg^-1^, and organic matter 38.7 g·kg^-1^.

### Experimental Design and Treatments

In the experiment, a 100 m^2^ area of soil was selected and divided into four plots for disinfectant experimental treatments, each with an area of 25 m^2^. The corn fields on the edge of the plots were used as a control treatment. The pepper variety used was Jufeng No.1. In this experiment, the venturi system was used for drip irrigation beginning with clear water for 15–30 min to make the water diffuse, followed by trial disinfectant for about 30–40 min (short for sandy soil and long for clay; short for shallow-rooted crops, and long for deep-rooted crops) so as to ensure uniform application. After the drip irrigation with trial disinfectant, drip irrigation with clear water continued for 15–30 min, and then the soil surface was covered with plastic film. After 3–5 days of film mulching, planting was carried out. The experiment was divided into five treatments, including four pesticide treatments and one control group (Table 1), which were randomly distributed. The first three treatments were treated with different dosages of 20% AITC (Zhongnong Qimin Science and Technology Development Co., Ltd., China) by the drip irrigation system fertilizer applicator, among which treatment 1 (AITC7, F) had 7 L/667 m^2^, treatment 2 (AITC9, G) had 9 L/667 m^2^, and treatment 3 (AITC11, H) had 11 L/667 m^2^. Treatment 4 (WP, I) was treated with 50% carbendazim (Shangbonong Plant Protection Co., Ltd., China) mixed with soil at a dosage of 2 kg/667 m^2^ as control disinfectant treatment, which was used for soil disinfection for local farmers, in order to form a comparison with AITC soil disinfection. Treatment 5 (CK) was the control without any treatment. Each treatment group included three duplicates.

### Soil Sampling

Soil sampling was conducted after planting for 30 d in each plot in March 2019. Rhizosphere soil was collected from pepper after excluding soil surface litter according to the “Z” shape sampling method in each plot, then mixed to make a single composite sample and divided into two parts, of which one was used for determining of soil enzyme activity while the second was stored at −20°C for DNA extraction.

### Analysis of Soil Enzyme Activity

The activities of sucrase and cellulose were determined by colorimetric method of 3,5-dinitrosalicylic acid with the reduction of 3,5-dinitrosalicylic acid to 3-amino-5-nitrosalicylic acid, while urease and catalase activity were assayed through the methods of indophenol blue colorimetry and potassium permanganate titration, respectively.

### Soil DNA Extraction, PCR Amplification, and High-throughput Sequencing

Total soil genomic DNA was extracted from 0.25 to 0.5 g of each soil sample using HiPure Soil DNA Kits (Magen, China) according to the manufacturer’s protocols. DNA was extracted above as a template, and the bacterial primers 341F [5’-CCTACGGGNGGCWGCAG-3’] and 806R [5’-GGACTACHVGGGTATCTAAT-3’] and fungal primers KYO2F [5’-GATGAAGAACGYAGYRAA-3’] and ITS4R [5’-TCCTCCGCTTATTGATATGC-3’] were used to amplify bacterial 16S rRNA genes in V3−V4 hypervariable regions and fungal genes in the ITS2 region by PCR, respectively. Amplicons were extracted from 2% agarose gels and purified using the AxyPrep DNA Gel Extraction Kit (Axygen Biosciences, USA) according to the manufacturer’s instructions and quantified using the ABI StepOnePlus Real-Time PCR System (Life Technologies, USA). Purified amplicons were pooled in equimolar and paired-end sequenced (2 × 250) on the Illumina Hiseq2500 PE250 platform according to the standard protocols. The raw reads were deposited into the NCBI Sequence Read Archive (SRA) database (Accession Numbers: PRJNA659124 and PRJNA659119).

### Bioinformatic Analysis

Because the raw data obtained contains some low-quality sequences and a disproportionate amount of N bases, which will affect the subsequent analysis, it is necessary to preprocess the raw data. The specific processing steps were as follows: raw data filtering, tag splicing, tag filtering, and tags de mosaicism, and high-quality optimized sequence data were obtained for subsequent information analysis. High-quality effective tags of 16S rRNA and ITS genes were obtained after quality control. Then, effective tags of the 16S rRNA and ITS genes were clustered with operational taxonomic units (OTUs) at 97% similarity levels using the UPARSE [40] pipeline and the tag sequence with highest abundance was selected as a representative sequence within each cluster. Venn analysis was performed in R project (version 3.4.1) to identify unique and common OTUs between groups. The representative sequences were classified into organisms by a naive Bayesian model using RDP classifier (version 2.2) [41] based on SILVA [42] and UNITE [43]. Biomarker features in each group were screened by Metastats (version 20090414)[44]. Chao1, Simpson, and all other alpha diversity indexes were calculated in Quantitative Insights Into Microbial Ecology (QIIME). OTU rarefaction curve and rank abundance curves were plotted in QIIME. Principal coordinates analysis (PCoA) was used to conduct multivariate statistical analysis in R project. Next, the microbial diversity and community structure of all soil samples were analyzed through taxonomy annotation and α and β diversity analysis. Finally, differential analysis was conducted using Metastats software to find biomarker features in each group.

### Statistical Analysis

Statistical analysis was conducted with the SPSS 22.0. The data were expressed as the means plus or minus one standard deviation from three replicates. The statistical significance of the differences between samples was determined by one-way analysis of variance (ANOVA) followed by Duncan’s multiple range test. *p* < 0.05 and *p* < 0.01 were considered significant differences and extremely significant differences. Alpha index comparison between groups was calculated by Welch’s t-test and Wilcoxon signed rank test in R project. The difference was considered statistically significant when the treatment group compared to the control group showed *p* < 0.05, and *p* < 0.01 was a highly significant difference. The relationship between soil enzymes and microbial diversity and abundance was analyzed by Pearson correlation coefficient and the correlation between soil enzyme activities and microbial community structure (bacterial and fungi) was analyzed by redundancy analysis (RDA).

## Results

### Effects of Different Treatments on Soil Enzyme Activities

After different doses of AITC treatments, changes in the activities of sucrase, catalase, urease, and cellulase were observed (Table 2). There was a significant increase in urease activity under AITC7, AITC9, and AITC11 fumigation by 7.6%, 29.0%, and 46.3%, respectively, compared to the control, on which the effect of AITC was less than that of WP. The effects of AITC and WP on catalase activity were similar to the control. Cellulase activity increased after AITC treatment and reached the maximum value of 1.86 mg/72 h·g in the AITC7 treatment, while WP significantly decreased the activity of cellulase. Additionally, AITC7 decreased the activity of sucrase, and with the increase of its concentration, its activity gradually increased and was significantly higher than that of the control, but the activity of this enzyme was significantly lower than that of WP. In conclusion, AITC and WP could increase the activities of urease, cellulase, and sucrase, but AITC had less effect than WP, whereas both treatments had little effect on catalase.

### Sequencing Data and Alpha Diversity Analysis

After removing low quality data, 1347518 and 1380109 effective bacterial and fungal tags, were obtained from the 15 soil samples respectively. The average lengths of bacterial and fungal sequences were 418 and 340 bp, respectively. A total of 13991 bacterial and 1258 fungal OTUs were generated by clustering.

Rarefaction curves can be used to evaluate whether the sequencing amount is sufficient to cover all taxa observed in the soil samples, which also indirectly reflects the species richness of the samples. When the curve tends to be flat or reaches the plateau stage, it can be considered that the sequencing depth has basically covered all of the species in the samples. The observed rarefaction analysis showed that the number of recorded OTUs of 16S genes and fungal ITS genes tended to approach saturation, which indicated that the sequencing data could reasonably estimate the bacteria and fungi taxa present in the soil samples (Fig. S1).

In order to identify whether different treatments were correlated with microbial community alteration, we profiled the divergence in richness and diversity of the microbial community in each sample. Alpha diversity indices were calculated using OTUs belonging to each sample based on the even depth of sampling (Table 3). The richness and diversity of microbial communities in soil were reflected by the α diversity indexes, such as Chao1, ACE, Goods Coverage, Observed Species, and Shannon and Simpson indexes. Different doses of AITC fumigation had different effects on the richness and diversity of soil bacterial and fungal communities (Table 3).

A total of 822,932 effective sequences were obtained in the bacterial community analysis of 15 soil samples. The Good’s Coverage values were higher than 98%, implying that there was sufficient sequencing depth for assessing the bacterial biodiversity in various soil samples. The observed OTU numbers in different treatments ranged from 4524 to 4787, of which the number was lower under the application of AITC treatments compared to the control and higher than in the WP treatment group, suggesting that AITC had significant effects on the richness of the bacterial community. AITC fumigation decreased bacterial diversity compared to the CK group and increased bacterial diversity compared to the treatment group with WP for the Chao1 and ACE indexes (Table 3), and the effects of different doses varied. According to the α indexes, the decrease of bacterial diversity did not show any trend with the increase of AITC doses. In addition, WP had more impacts than AITC for the Chao1 and ACE indexes.

The number of OTUs observed in fungi was significantly lower than that in bacteria, and fungal diversity was increased by AITC, especially in treatments AITC7 and AITC11, compared to the CK and WP treatment groups for Chao 1 and ACE. The fungal diversity of the AITC9 treatment group was lower than that of the control group, but larger than the WP treatment group. In conclusion, AITC also significantly affected fungal diversity and richness (Table 3).

There is a certain degree of similarity and specificity in the species distribution of microbial communities in different habitats and venn diagrams are useful for quantifying and visualizing the number of common and specific OTUs between groups or samples. There were 2253 bacterial OTUs (Fig. S2A) shared by the treatment groups AITC7, AITC9, AITC11, WP, and CK, and the number of unique OTUs in each group were 611, 669, 1571, 1005, and 987, respectively. There were 229 fungal OTUs (Fig. S2B) shared by treatment groups AITC7, AITC9, AITC11, WP, and CK, and the number of unique OTUs in these groups were 163, 71, 53, 48, and 75, respectively. These results suggested that there were some differences in the effects of different treatments on microorganisms in soil.

### Bacterial and Fungal Community Structure Analysis

**Fig. 1:**
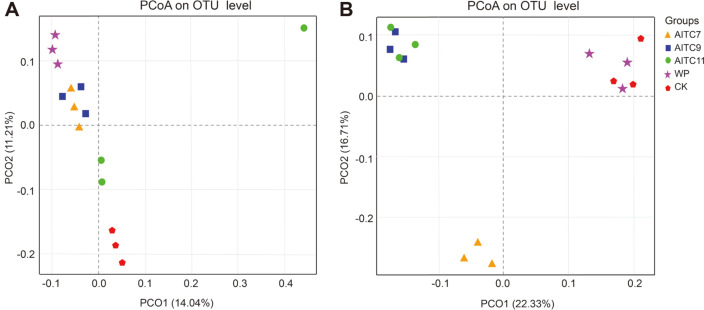
Principal coordinates analysis (PCoA) of 16S rRNA gene bacterial communities (A) and ITS gene fungal communities (B) from the five treatments. Each colored and shaped dot represents a sample.

Variations in the microbial community of AITC-treated and non-treated samples were evaluated using principal coordinates analysis (PCoA), in which the horizontal (PC1 axis) and vertical coordinates (PC2 axis) were the principal coordinate components contributing to the differences in the soil microbial bacterial (Fig. 1A) and fungal (Fig. 1B) community composition among all samples.

The total variance contribution rate axis to the difference of bacterial community composition of samples was 25.25% (PCO1, 14.04% and PCO2, 11.21%) (Fig. 1A), indicating that AITC dosage was the major influencing factor that contributed to the difference in bacterial community composition between all samples. The distance between the samples of each group was close, suggesting that repeatability between samples of the same group was high and that the difference between the samples of each group was not very large (Fig. 1A). Fungal communities were significantly separated between the control group and the other treatment groups in the samples, in which CK and WP groups, AITC7 group, and AITC9 and AITC11 groups were distributed in the first, third, and second quadrants, respectively (Fig. 1B). The short distance between the CK and WP groups indicated that the difference between them was small, while the difference between the two groups and the other AITC treatment groups was large. In addition, it can be seen from the PcoA of bacteria that compared with the CK, the differences between the low-dose and medium-dose AITC treatment were greater than those between the high-dose treatment and the control, but for fungi, the differences between medium-dose and high-dose AITC treatments were small, while the difference between the low-dose treatment and the medium- and high-dose treatments was large. The variance contribution rates of the PCo1 axis and PCo2 axis to the fungal community composition of samples were 22.33 and 16.71%, respectively. The variance rate of fungi was higher than that of bacteria, suggesting that the effects of AITP on fungal community composition were greater than on bacteria.

**Fig. 2:**
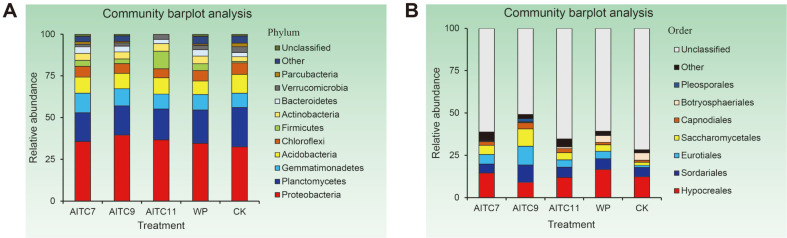
The 16S rRNA gene-based bacterial community compositions at the phylum level (A) and ITS genebased fungal community compositions at the order level (B) compositions in five treatments.

The relative abundance accumulation map of species of the top 10 phyla in the bacterial community and the top 7 orders in the fungal community (relative abundance greater than 2%) in the samples were generated based on the results of gene sequencing analysis. The relative abundance of bacterial phyla and fungal orders is shown by bar plots in Fig. 2.

The dominant bacterial phyla in the bacterial community were Proteobacteria, Plantomycetes, Gemmatimonadetes, Acidobacteria, Chloroflexi, *Firmicutes*, Actinobacteria, Bacteroidetes, Verrucomicrobia, and Parcubacteria (Fig. 2A). Compared to CK, the relative abundance of Gemmatimonadetes, *Firmicutes*, and Actinobacteria increased significantly after AITC fumigation, while that of Plantomycetes, Acidobacteria, Verrucomicrobia, and Parcubacteria decreased significantly. Plantomycetes decreased after AITC treatment compared with samples treated with WP, and Acidobacteria increased. With the increase of AITC dose, Proteobacteria first increased and then decreased, while Acidobacteria, *Firmicutes*, and Parcubacteria showed a tendency of decrease firstly and then increase. However, Plantomycetes, Actinobacteria, and Verrucomicrobia gradually increased and Gemmatimonadetes, Chloroflexi, and Bacteroidetes showed a downward trend in the AITC7, AITC9, and AITC11 treatments.

The top phyla with the highest relative abundance in the fungal community were Ascomycota and Basidiomycota, and the relative abundance of Ascomycota increased in treatments AITC7 and AITC9 and decreased in AITC11 treatment compared to CK, whereas it was higher in the three treatments of AITC7, AITC9, and AITC11 than treatment WP, of which maximum abundance was in the treatment AITC9. The change of the relative abundance of Basidiomycota was not obvious, but it increased in AITC7 and AITC11 and decreased in AITC9 compared with CK and WP (Fig. S3). The dominant orders of fungi in the fungal community were Hypocreales, Sordariales, Eurotiales, Saccharomycetales, Capnodiales, Botryosphaeriales, and Pleosporales (Fig. 2B). AITC fumigation significantly changed the community composition of fungi in soil. More obviously, the relative abundances of Eurotiales, Saccharomycetales, and Capnodiales were higher than the CK, while that of Botryosphaeriales decreased significantly compared to CK after AITC treatment. Compared to the treatment group with WP, the relative abundance of Hypocreales and Botryosphaeriales decreased significantly and that of Saccharomycetales and Capnodiales increased significantly after AITC fumigation, of which the maximum value occurred in the AITC9 group. Additionally, we found that some unclassified fungal orders decreased significantly after fumigation compared to CK. The functions and characteristics of these orders have not been determined yet.

**Fig. 3:**
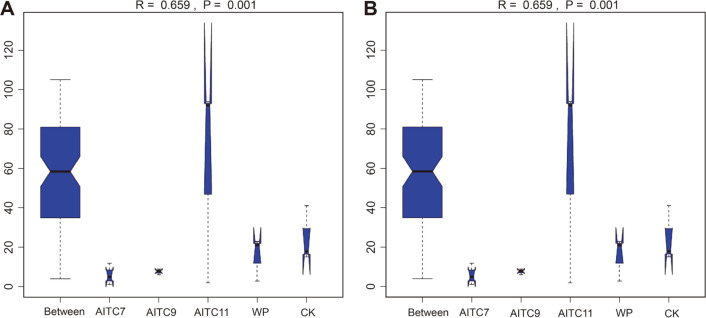
Differences analysis of ANOSIM of 16S rRNA gene-based bacteria (A) and ITS gene-based fungi (B) between the five treatments. *R*-value representes the degree of difference, generally between (0,1), among which *R* > 0 representes that differences between treatments. *R* > 0.75 means a big difference; *R* > 0.5 means a moderate difference; *R* > 0.25 means a small difference. When R is equal to or near 0 (when R is low than zero, they are considered invalid data), it indicates that there is no difference between groups. The reliability of statistical analysis is expressed by *p*-value, and *p* < 0.05 means that there are significant differences.

Analysis of similarity (ANOSIM) is a non-parametric test method for microbial community structure that is used to test whether the difference between groups is significantly greater than the difference within groups so as to determine whether the grouping is meaningful. Fig. 3 showed the values of *R* = 0.659, *p* = 0.001 for bacteria (Fig. 3A) and *R* = 0.815, *p* = 0.001 for fungi (Fig. 3B) among each group, indicating that there were significant differences in bacteria and fungi between groups.

### Significance Analysis of Species Differences among Five Treatments at the Genus Level

**Fig. 4:**
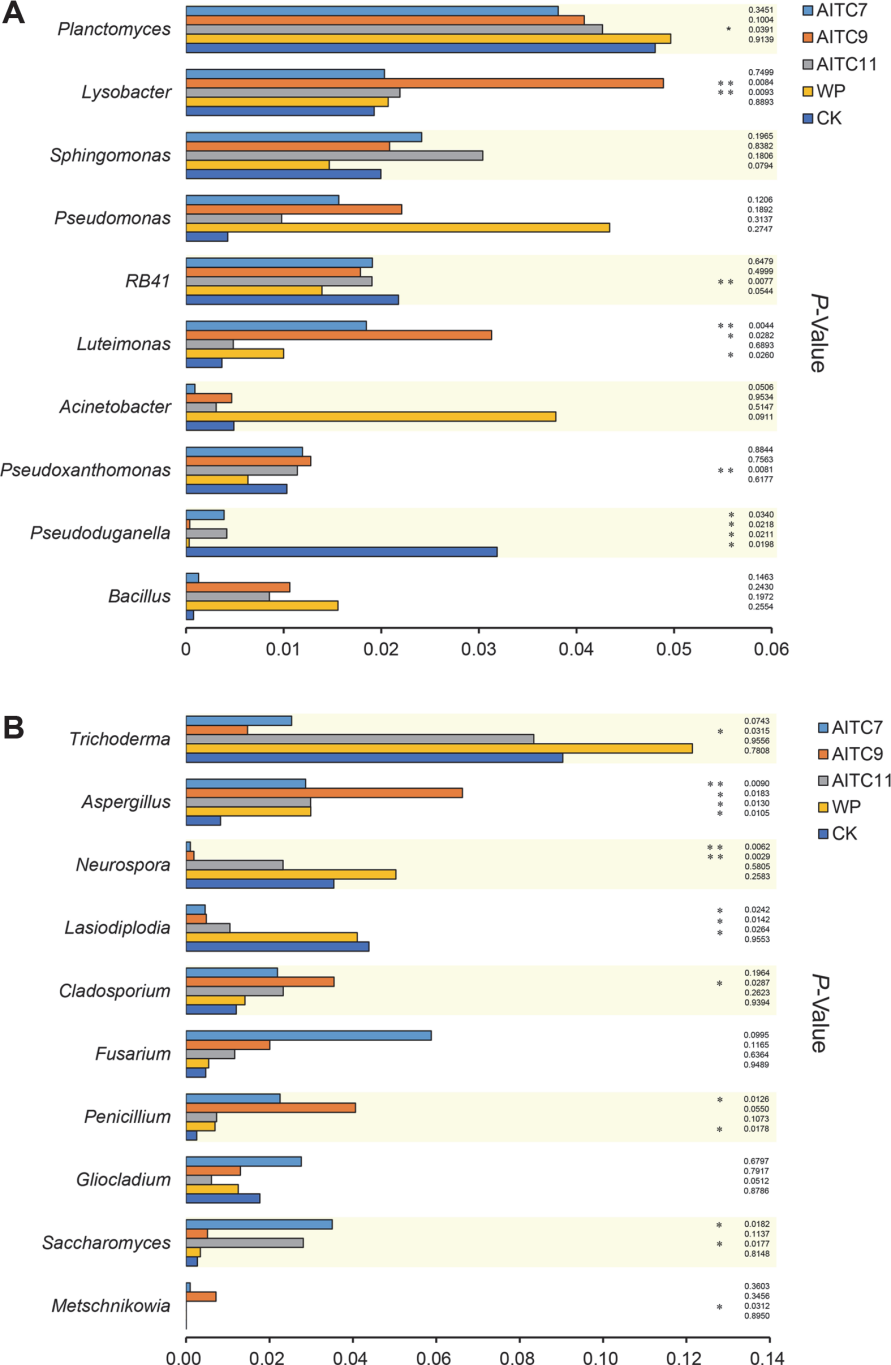
Statistically significant analysis of 16S rRNA gene-based bacterial (A) and ITS gene-based fungal (B) communities differences in genera in five treatments (*p < 0.05, **p < 0.01).

A statistical method utilizing Metastats software was used to test the difference of relative abundance of the top 15 genera of microbial communities between the two groups of samples at the genus level. The bacterial (Fig. 4A) and fungal (Fig. 4B) genera with significant differences between every treatment group and control group were obtained.

In the bacterial community, there were some significant differences in the relative abundances of bacterial genera. *Planctomyces* decreased significantly after AITC fumigation compared with CK, especially in the AITC11 treatment (*p* < 0.01) and became the most abundant bacterial genus in this treatment, while AITC reduced the relative abundance ratio of *RB41*, which was extremely significant in AITC11 treatment (*p* < 0.01). After AITC treatment, the relative abundance of *Sphingomonas*, *Pseudomonas*, and *Bacillus* increased and that of *Acinetobacter* was lower than CK, but there was no significant difference in the samples. *Luteimonas* also significantly increased after AITC treatments, especially in the treatment groups AITC7 (*p* < 0.01) and AITC9 (*p* < 0.05). Compared to CK, the relative abundance of *Pseudoxanthomonas* in treatment AITC11 decreased remarkably following AITC treatment, at a rate 1.1 times higher than that in the control. In addition, the application of AITC significantly reduced the relative abundance of *Pseudoduganella*, which had nothing to do with the amount added. The relative abundance of bacteria was also affected by WP compared to CK treatment; *Planctomyces*, *Lysobacter*, *Pseudomonas*, *Luteimonas*, *Acinetobacter*, and *Bacillus* increased and *Sphingomonas*, *RB41*, *Pseudoxanthomonas*, and *Pseudoduganella* decreased, but a significant difference was only present in *Luteimonas* and *Pseudoduganella*. In conclusion, AITC treatment, such as AITC11, had the greatest effect on bacteria (Fig. 4A).

The effect of AITC on fungi was less than that on bacteria. At first, AITC fumigation reduced the relative abundance of some fungal genera. For example, *Trichoderma* (*p* < 0.05 in the AITC9 treatment), *Neurospora* (*p* < 0.01 in the AITC7 and AITC9 treatments), and *Lasiodiplodia* (*p* < 0.05 in all AITC treatments) were reduced, while there was a non-significant increase in the abundance of *Fusarium*. For *Gliocladium*, the relative abundance was reduced by the application of AITC7, but it decreased with the increase of AITC compared with the control group. The relative abundance of *Penicillium* also increased with the increase of the AITC, and there was a significant difference in the AITC7 treatment. The relative abundance of *Saccharomyces* was increased by AITC fumigation, and there were significant differences (*p* < 0.05) in the treatments with low doses and high doses of AITC. AITC treatment had the strongest effects on the relative abundance of *Aspergillus*, with significant differences found in the AITC9 and AITC11 treatments and a highly significant difference in the AITC7 treatment. The WP treatment significantly affected the relative abundance of *Aspergillus* and *Penicillium*, but had little effect on other genera (Fig. 4B).

### Correlation Analysis Between Soil Enzyme Activity and Microbial Diversity

The Shannon index and OTUs were used to analyze the correlation between soil enzyme activity and microbial diversity. Table 4 showed that urease was positively correlated with fungal diversity index, but negatively correlated with bacterial diversity index, bacterial OTUs, and fungal OTUs, and significantly negatively correlated with fungal OTUs. Catalase was positively correlated with bacterial diversity and OTU number, but negatively correlated with fungal diversity and OTUs. Except for the bacterial diversity index, cellulase was positively correlated with others, and was significantly correlated with fungal OTUs. On the contrary, sucrase was only positively correlated with fungal diversity index, but negatively correlated with fungal OTUs. In conclusion, the correlation between the four enzymes and fungal OTUs was obvious.

### Relationships Analysis Between Soil Enzymes and Primary Genus

**Fig. 5:**
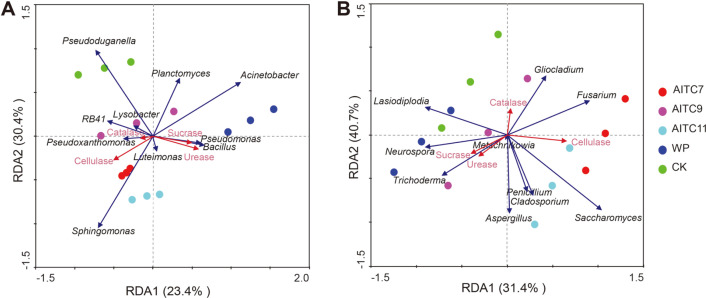
Redundancy analysis (RDA) of the total (A) bacterial (B) fungal communities and soil enzymes for individual samples. Soil enzymes included sucrase, catalase, urease and cellulase. The direction of the arrows indicates correlations with the first two canonical axes, and the length of the arrows represents the strength of the correlations. In all treatments, AITC7 had a rate of 7 L/667 m^2^ 20% AITC; AITC9 had a rate of 9 L/667 m^2^ 20% AITC; AITC11 had a rate of 11 L/ 667 m^2^ 20% AITC; WP had a rate of 2 kg/667 m^2^ 50% carbendazim; CK was the control. Each group included three replicates.

Based on the previous research results redundancy analyses (RDA) between the important soil enzymes and the top 10 genera (including bacteria and fungi) were conducted (Fig. 5). The results showed that four important soil enzymes explained 53.8% and 72.1% of total variability for bacterial (Fig. 5A) and fungal (Fig. 5B) community composition, respectively.

Fig. 5A shows that *Pseudomonas*, *Bacillus*, *Acinetobacter*, and *Luteimonas* were positively correlated with sucrase and urease, and negatively correlated with catalase and cellulose. In contrast, some genera such as *RB41*, *Pseudoxanthomonas*, and *Sphingomonas* presented negative correlations with sucrase and urease, but positive correlations with cellulase and catalase.

Similarly, it was seen in Fig. 5B that some genera of fungi, such as *Trichoderma*, *Neurospora*, and *Lasiodiplaplodia*, were positively correlated with sucrose and urease; catalase was positively correlated with *Gliocladium* and *Fusarium*; and cellulase was positively correlated with *Saccharomyces* and *Fusarium*. These enzymes were not only positively correlated with fungi, but there were also a negative correlation and no correlation.

## Discussion

Soil fertility status is reflected by the activity of microorganisms, which plays a key role in maintaining the stability of soil ecosystems and the biological process of soil productivity formation [45]. If microbial life is disrupted, soil fertility and plant health will also be adversely affected. AITC plays an important role in agricultural biological control [46]. Therefore, it is of great significance to study the effect of AITC on soil microorganisms.

In this study, we found that AITC fumigation had significant effects on the richness and diversity soil microorganisms compared to CK at both high and low dosages (Table 3). Bacterial diversity significantly decreased following AITC fumigation, which was consistent with the results of a previous study [1]. AITC reduced the richness and diversity of bacteria (Table 3) and the effect of a low dose of AITC was greatest, which was inconsistent with the results of another study [30] that found that AITC had no significant effect on soil bacterial richness, but only temporarily inhibited the diversity of bacterial phyla within the community. This may be related to the sampling time, the dosage of AITC, and the plant species used, because AITC can degrade and the impact on microorganisms may be temporary [47]. The inhibitory effect of high-dose AITC was also reported in literature [35] in tomatoes. Unlike the bacterial community, AITC with different doses stimulated fungal richness and significantly increased fungal community diversity (Table 3), which was similar to the effect of MS fumigation [30]. Our result was contrary to the study of Zhu *et al*. [1], in which AITC inhibited the abundance of fungi in soil, possibly due to fact that tomato plants and some fungi in tomato soil were sensitive to AITC. For example, after fumigation, the relative abundance of *Trichoderma*, the dominant fungus in our study, decreased significantly, especially in the medium-concentration AITC, while other fungi increased, possibly due to an increase in the available breeding space and competitiveness of the other fungi [1].

Through the analysis of beta-diversity, PCoA showed that AITC fumigation had less effect on soil bacterial community structure than on soil fungal community structure according to the total variance contribution rate (Fig. 1A), which was consistent with a previous study [1], in which the dominant bacteria were mostly the same, while the dominant fungi showed a large difference, indicating that microbial beta-diversity could be related to AITC dosage, plant species, and other factors. According to this study and others’ research [1, 30], a conclusion can be drawn that AITC fumigation has a greater impact on microorganisms than plants, and its effects on fungal community structure are greater than bacteria regardless of the plant species. Moreover, we found that AITC application led to the change of bacteria in AITC treatments compared with CK, but the abundance of different bacteria varied with the change of AITC dose (Fig. 4A). At the bacterial genus level, the relative abundance of *Pseudomonas*, *Bacillus*, and *Sphingomonas* increased after AITC fumigation. These are considered to be beneficial bacteria in soil because of their degradation function and ability to decompose organic pollutants and release antibiotics in soil [48, 49]. The relative abundance of *Lysobacter* was also increased. This genus of bacteria has bacteriolytic functions against many pathogenic fungi, bacteria, and nematodes, and can secrete a variety of antibiotics [50]. This is consistent with the previous studies that AITC improves disease resistance.

In the fungal community (Fig. 4B), as important growth-promoting fungi in soil, *Trichoderma* can promote the growth and development of crops by mineralization of organic matter and other mechanisms [51], inhibit the growth of pathogens, and prevent and control soil-borne diseases by producing antibiotics such as antimicrobial peptides [11]. The results showed that AITC was not conducive to the reproduction of this strain. When infected with *Botrytis cinerea*, *Gliocladium* can reproduce and eventually occupy the parasite infection site [51]. Moreover, *Gliocladium* can also produce related enzymes and bioactive secondary metabolites, and so can be used as broad-spectrum biological control to control a variety of plant pathogenic fungi by destroying the cell walls of the pathogen and causing the pathogen to die and *Gliocladium* is a universal fungicide [52]. The relative abundance of *Gliocladium* increased following the application of low doses of AITC, and decreased with the increase of AITC concentration, indicating that low-dose AITC can promote the activity of this strain and achieve the effect of disease resistance. However, the relative abundance of degrading bacteria, *Aspergillus*, and *Penicillium* were also increased, while the effect of high-dose AITC was poor. *Cladosporium* is also a microorganism that can degrade organic matter [53]. These are beneficial bacteria in soil that play important roles in soil biological control. AITC fumigation can increase the diversity of the soil fungal community, optimize the structure of the soil fungal community, and produce a large number of beneficial flora, which can effectively control soil-borne diseases and create a relatively healthy microbial ecological environment in the soil [1]. It was found that AITC could degrade rapidly in soil, which is a useful feature because it will produce toxicity to microorganisms if it is present in soil for long periods of time.

It has been found that AITC fumigation had a fertilization effect similar to CP, which may be caused by the increase in the number of beneficial microbial communities in the soil [1]. For example, Ren *et al*. found that AITC could promote the growth of tomato plants after fumigating the soil [17]. However, a number of studies have found that the relative abundance of some microorganisms was related to the concentration and timing of AITC fumigation [30]. For example, AITC fumigation significantly increased the relative abundance of *Bacillus* only on the second day of incubation and then returned to control levels, while high doses of AITC inhibited the diversity and richness of bacterial community for a long time, although they showed a trend of recovery [1]. *Saccharomyces* and *Metschnikowia* are two yeasts that play key roles in the decomposition of organic matter and can also release antibodies [54]. Our research found that the relative abundance of these two kinds of yeast increased significantly compared with the control group and the control agent treatment, indicating that AITC can increase the abundance of beneficial microorganisms, further improve soil fertility, and promote plant growth [55]. *Acinetobacter* is a common bacteria in soil. It has been found that *Acinetobacter* can degrade the sulfur and phosphorus of pesticides, which can repair soil polluted by pesticides, and some species have degradation functions [56]. In our study, we found that the abundance of *Acinetobacter* species decreased after AITC fumigation, which may be related to their short life cycles [57]. Although *Fusarium* is a typical pathogen [58], its relative abundance increased when it was treated with AITC. However, some studies have found that some species of *Fusarium* were non-pathogenic strains or weakly parasitic fungi that can be used for biological control, indicating that AITC can make *Fusarium* produce disease resistance. In addition, studies have speculated that some *Fusarium* and AITC may cooperate to control nematodes and pathogens, of which the decrease in abundance caused by high doses of AITC can be caused by stronger competition among fungi [59].

Soil enzyme activity is the embodiment of soil microbial functional diversity, and is the direct expression of soil biological community metabolism and available nutrients [60]. Our study found that soil enzyme activities were increased by AITC (Table 2) and correlated with microbial diversity (Table 4), the relative abundance of dominant microbia (including bacteria and fungi), and the distribution of soil samples in different treatments (Fig. 5), indicating that soil enzyme activity may be related to microbial activity. Mungai *et al*. [61] found that the functional diversity of soil microorganism was critical to the activity of soil enzymes. Research findings have also shown that the functional diversity of soil microorganism was critical to the activity of soil enzymes and that *Pythium* and *Trichoderma* increased the activities of acid and alkaline phosphatase, urease, cellulolytic enzyme, β-glucanase, and chitinase related to C, N, and P cycles in sandy loam soil [62]. Dempsey *et al*. also found that inoculation significantly changed the soil microbial community, resulting in a significant increase in soil glucosidase and cellulase activities [63]. Interestingly, we found that AITC can reduce the diversity of bacteria and increase the diversity of fungi at a certain concentration, which is consistent with the previous results, and the generated results may be related to the enzymes secreted by fungi. Fungi can secrete some enzymes, such as extracellular oxidoreductase and hydrolase, that can be metabolized by other microorganisms [45, 46], and the secondary products of AITC can be used as nutrients by the metabolism of fungi to increase microbial richness and diversity [1], which not only demonstrates that enzyme activities are related to microbial activity, but also shows that AITC can significantly affect microbial diversity. More importantly, compared with WP, AITC can increase the diversity of microorganisms, which indicates that AITC has certain advantages and can promote the reproduction of microorganisms [66].

We only studied the effect of AITC fumigant on microorganisms in continuous cropping pepper soil, but the effect on uncultivated soil has not yet been revealed. If we research the effects of AITC on microorganisms in the soil without planting for microbial diversity analysis and comparison, this may better reveal the effect of AITC because plants can also affect microorganisms to a certain extent [67]. More importantly, AITC is an unstable and easily degradable sulfur compound that can be decomposed into various secondary products through biochemical reactions under certain conditions, which may be related to microbial metabolism [68]. If we combine soil metabolism with the degradation of AITC in soil, we may reveal more details on the relationship among AITC, soil, and microorganisms.

In short, through investigating the effects of AITC on soil microbial diversity and community composition in this study, we found that microbial diversity and community structure were significantly influenced by different dosages of AITC, among which the effect on soil bacteria was less than that on fungi. AITC reduced the diversity of soil bacteria, stimulated the diversity of the soil fungal community, and significantly changed the structure of the fungal community. AITC reduced the relative abundance of dominant bacteria *Planctomycetes*, *Acinetobacter*, *Pseudodeganella*, and *RB41*, but increased that of *Lysobacter*, *Sphingomonas*, *Pseudomonas*, *Luteimonas*, *Pseudoxanthomonas*, and *Bacillus* at the genera level. Among fungi, the relative abundances of *Trichoderma*, *Neurospora*, *Trichoderma*, *Neurospora*, and *Lasiodiplodia* decreased significantly and that of *Aspergillus*, *Cladosporium*, *Fusarium*, *Penicillium*, and *Saccharomyces* were higher than the control. Additionally, the relative abundance changes of other genera were not consistent, which was related to AITC dosage. The correlations between soil enzyme and microbial diversity and abundance indicated that soil enzymes had a strong correlation with fungal OTUs, and cellulase had a significant correlation with fungal OTUs. The correlation between soil enzymes and the microbial community showed that catalase and cellulase were key enzymes affecting the bacterial community, while the fungal community was correlated with sucrase and urease. Our results provide useful information for the use of AITC in the assessment of environmental and ecological security.

## Supplemental Materials

Supplementary data for this paper are available on-line only at http://jmb.or.kr.

## Figures and Tables

**Table 1:** Experimental design and treatment management.

Treatment serials	Treatment number	Treatment description
1	AITC7	20% AITC (7 L / 667 m^2^)
2	AITC9	20% AITC (9 L/667 m^2^)
3	AITC11	20% AITC (11 L/667 m^2^)
4	WP	50% Carbendazim (2 kg/667 m^2^)
5	CK	without any treatments

**Table 2:** Effects of different treatments on soil enzyme activities in pepper soil.

Treatments	Urease (mg/g·24 h)	Catalase (mg/g·min)	Cellulase (mg/72 h·g)	Sucrase (mg/g·24 h)
AITC7	46.18 ± 0.41b	0.20 ± 0.02a	1.86 ± 0.04d	2.32 ± 0.12a
AITC9	55.41 ± 0.03c	0.24 ± 0.05a	1.42 ± 0.02b	3.94 ± 0.04c
AITC11	62.80 ± 0.70d	0.24 ± 0.03a	1.65 ± 0.04c	4.46 ± 0.03d
WP	72.15 ± 0.06e	0.23 ± 0.03a	0.92 ± 0.03a	5.02 ± 0.01e
CK	42.94 ± 0.07a	0.23 ± 0.04a	1.42 ± 0.01b	2.82 ± 0.01b

^a^Values are mean ± standard deviation (*n* = 3).

^b^Different letters in the same column represent significant differences at the *p* = 0.05 level.

**Table 3:** Effects of different treatments on coverage, observed OTU numbers, richness, and diversity.

Microbial species	Treatments	Chao1	ACE	Goods Coverage	Observed Species	Shannon	Simpson
Bacterial	AITC7	5291.322±195.5135 a	5365.018±180.0468 bc	0.98504±0.0029 bc	4524±129.7421 a	9.843741±0.2671 a	0.995986±0.0012 a
	AITC9	5378.351±194.8603 bc	5419.588±230.5469 bc	0.985747±0.0011 bc	4590±192.6664 a	9.822586±0.1752 a	0.995646±0.0010 a
	AITC11	5338.934±89.7268 bc	5453.663±122.7499 bc	0.983449±0.0016 a	4658±204.1348 a	9.853658±0.3087 a	0.99441±0.0038 a
	WP	5227.108±150.1879 a	5229.829±194.2926 a	0.987639±0.0024 c	4536±113.6148 a	9.875996±0.5627 a	0.992098±0.0082 a
	CK	5589.578±42.2844 d	5658.038±90.2645 c	0.983994±0.0015 bc	4787±68.2373 a	10.01664±0.2612 a	0.994771±0.0033 a

Fungi	AITC7	684.4263±47.2747 d	691.2801±34.8127 d	0.998561±0.0001 ab	562±36.3868 d	5.546285±0.2072 a	0.939937±0.0129 a
	AITC9	571.3056±48.9797 bc	583.145±54.6466 bc	0.99881±0.0001 b	487±47.5219 bc	5.24054±0.2424 a	0.930165±0.0153 a
	AITC11	644.6144±48.2518 cd	651.6884±42.3382 cd	0.998521±0.0002 a	507±17.0098 cd	5.082764±0.8319 a	0.906041±0.07840 a
	WP	547.0972±38.1086 a	548.7504±36.2494 a	0.998745±0.0001 ab	432±22.0076 a	5.218259±0.3357 a	0.942193±0.0178 a
	CK	583.895±15.2950 bc	602.3794±12.0693 bc	0.998763±0.0001 ab	481±19.6977 bc	4.57886±0.8487 a	0.88007±0.0929 a

^a^Values are mean ± standard deviation (*n* = 3).

^b^Different lower case letters in the same column indicate significant differences, while the same lower case letters indicate insignificant differences (*p* < 0.05).

**Table 4:** Correlation between soil enzyme activity and microbial diversity.

Soil enzyme	Bacterial Shannon index	Fungal Shannon index	Bacterial OTUs	Fungal OTUs
Urease	-0.069	0.127	-0.287	-0.533*
Catalase	0.106	-0.307	0.264	-0.308
Cellulase	-0.041	0.179	0.068	0.834**
Sucrase	-0.103	0.001	-0.133	-0.620*

^a^means *p* < 0.05 and **means *p* < 0.01 (*n* = 3).
