# Supplementary material for: Influence of Allyl Isothiocyanate on the Soil Microbial Community Structure and Composition during Pepper Cultivation
Source: J Microbiol Biotechnol. 2021 May 29;31(7):978–89. doi: 10.4014/jmb.2012.12016 (PMC9705941; doi:10.4014/jmb.2012.12016)
Supplement: Supplementary file 1 [file jmb-31-7-978-supple.pdf]

J. Microbiol. Biotechnol.
https://doi.org/10.4014/jmb.2012.12016

Supplementary file

Influence of allyl isothiocyanate on the soil microbial community structure and composition during pepper cultivation

Jingxia Gao[1], Hongxia Pei[1], Hua Xie[1]*

[1] Institute of Germplasm Resources, Ningxia Academy of Agriculture and Forestry Sciences, Yinchuan, 750002, China

*Correspondence

Hua Xie

Institute of Germplasm Resources, Ningxia Academy of Agriculture and Forestry Sciences, Yinchuan 750002, China

E-mail: 774350762@qq.com.

Figure captions:

Figure S1 Rarefaction curves of 16S rRNA gene-based bacteria (a) and ITS gene-based fungi (b).

Figure S2 Venn diagrams of comparison of bacterial (a) and fungal (b) OTUs in all five treatments.

Figure S3 Relative abundances of the fungal communities at the phylum level in five treatments.

In all of the treatments, AITC7 had a rate of 7 L/667 m2 20% AITC; AITC9 had a rate of 9 L/667 m2 20% AITC; AITC11 had a rate of 11 L/667 m2 20% AITC; WP had a rate of 2 kg/667 m2 50% carbendazim; and CK was the control. Each group included three replicates.

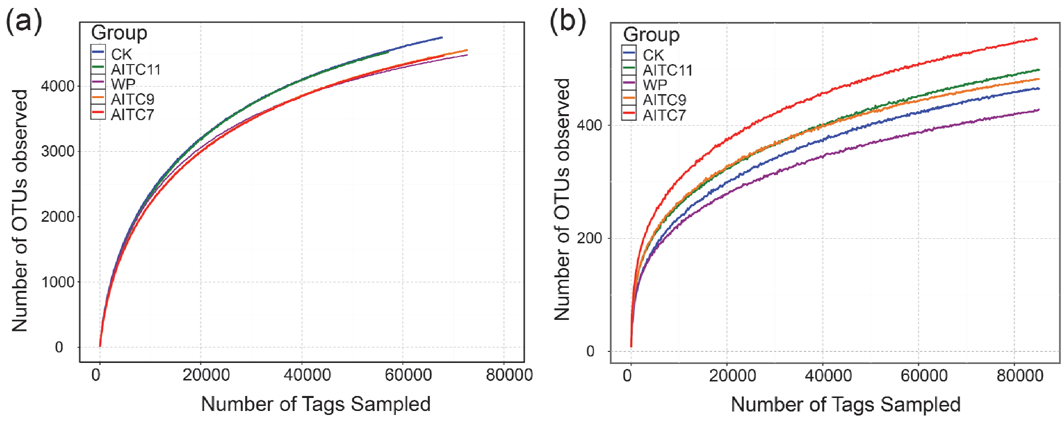
(a)
Group
CK
AITC11
WP
AITC9
AITC7
Number of OTUs observed
0
1000
2000
3000
4000
0
20000
40000
60000
80000
Number of Tags Sampled
(b)
Group
CK
AITC11
WP
AITC9
AITC7
Number of OTUs observed
0
200
400
0
20000
40000
60000
80000
Number of Tags Sampled

Figure S1

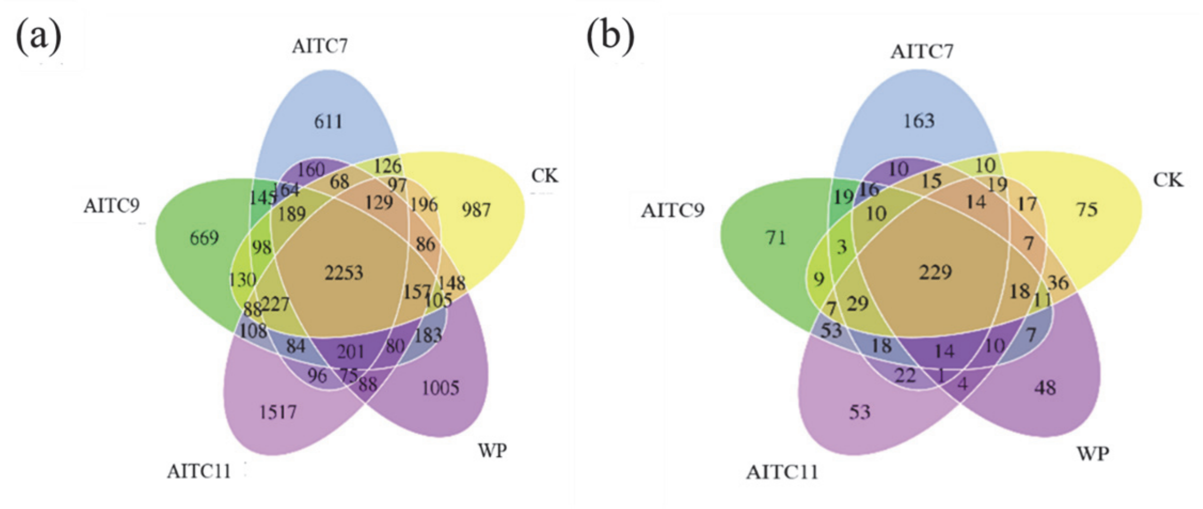
(a)
AITC7
611
160
126
CK
987
AITC9
669
145
164
68
97
189
129
196
98
86
2253
130
157
148
88
227
105
108
84
201
80
183
96
75
88
1005
1517
AITC11
WP
(b)
AITC7
163
10
10
CK
75
AITC9
71
19
16
15
19
10
14
17
3
7
229
9
18
36
7
29
11
53
18
14
10
7
22
1
4
48
53
AITC11
WP

Figure S2

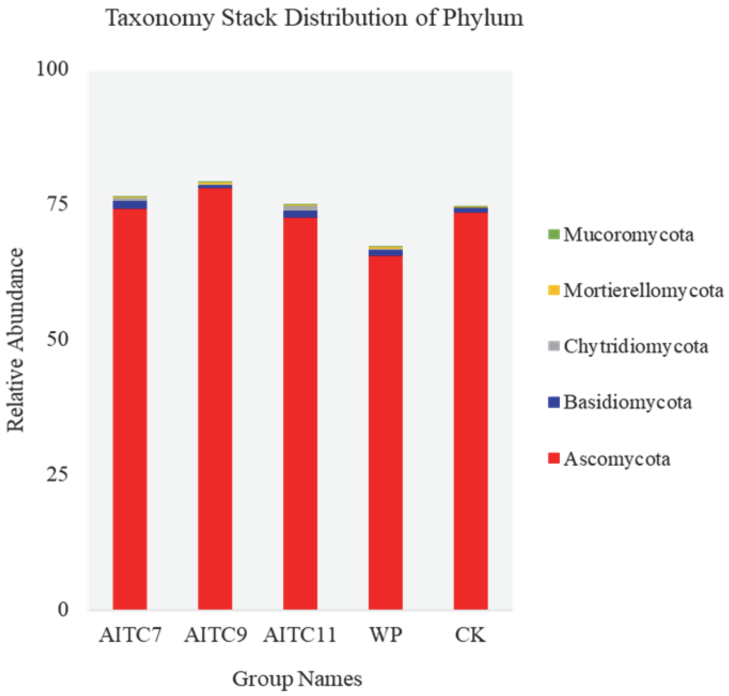
Taxonomy Stack Distribution of Phylum
100
75
50
25
0
Relative Abundance
AITC7
AITC9
AITC11
WP
CK
Group Names
Mucoromycota
Mortierellomycota
Chytridiomycota
Basidiomycota
Ascomycota

Figure S3
